# Consumption of dairy products and odds of ulcerative colitis: An Iranian case–control study

**DOI:** 10.1002/fsn3.3846

**Published:** 2023-11-20

**Authors:** Mohammad Reza Amini, Zeinab Khademi, Marieh Salavatizadeh, Zahra Kalantar, Nasser Ebrahimi‐Daryani, Ahmad Esmaillzadeh, Azita Hekmatdoost

**Affiliations:** ^1^ Student Research Committee, Department of Clinical Nutrition and Dietetics, Faculty of Nutrition Sciences and Food Technology, National Nutrition & Food Technology Research Institute Shahid Beheshti University of Medical Sciences Tehran Iran; ^2^ Department of Clinical Nutrition & Dietetics, National Nutrition & Food Technology Research Institute Shahid Beheshti University of Medical Sciences Tehran Iran; ^3^ Department of Public Health Sirjan School of Medical Sciences Sirjan Iran; ^4^ Department of clinical Nutrition, School of Nutritional Sciences and Dietetics Tehran University of Medical Sciences Tehran Iran; ^5^ Department of Gastroenterology and Hepatology Tehran University of Medical Sciences Tehran Iran; ^6^ Obesity and Eating Habits Research Center, Endocrinology and Metabolism Molecular – Cellular Sciences Institute Tehran University of Medical Sciences Tehran Iran; ^7^ Department of Community Nutrition, School of Nutritional Sciences and Dietetics Tehran University of Medical Sciences Tehran Iran

**Keywords:** case–control, dairy products, milk, ulcerative colitis

## Abstract

The association between dairy product consumption and the risk of ulcerative colitis (UC) is not well elucidated. This case–control study examined the association between Iranian adults' dairy consumption and UC risk. We used a valid food frequency questionnaire to analyze dietary intakes in 340 patients with pathologically confirmed cases of UC and 782 controls as part of a case–control research. Pasteurized milk, cheese, and yogurt dietary intakes were calculated along with dairy products. Other variables were acquired using questionnaires. Study participants' mean (± SD) age and body mass index were 41.5 ± 14.1 years and 27.4 ± 4.77 kg/m^2^, respectively. After adjusting for potential variables, individuals who consumed more total dairy products were less likely to get UC than those who consumed less (odds ratio [OR]: 0.44; 95% confidence interval (CI): 0.24, 0.79). We found a significant reverse association between milk intake (OR: 0.13; 95% CI: 0.07–0.24) and yogurt intake (OR: 0.52; 95% CI: 0.29–0.91) and UC, after controlling for potential confounders. Also, no significant association was found between cheese and UC risk (OR: 1.38; 95% CI: 0.84–2.28). Higher consumption of total dairy products may reduce UC risk. To be specific, milk and yogurt are inversely associated with this disorder. However, no link was found between cheese intake and UC. Longitudinal observational studies, especially cohorts, are needed to further assess these associations.

## Graphical abstract

Higher consumption of total dairy products may reduce UC risk. To be specific, milk and yogurt are inversely associated with this disorder. No significant association was found between cheese and UC risk.

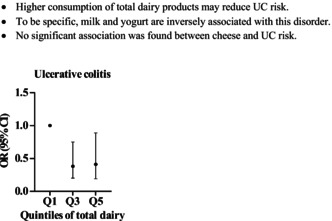

## 1 INTRODUCTION

Ulcerative colitis (UC) is a chronic, idiopathic, complex, and frequent inflammatory bowel disease (IBD), characterized by a periodically recurrent and relapsing mucosal inflammation course (Magro et al., 2017). In both developed and developing nations, the incidence and frequency of UC have dramatically increased recently (Kuo et al., 2015; Ramos & Papadakis, 2019). The reasons for this increasing trend in prevalence of this disease in the developing world are not clear; however, improvements in general health, exposure to certain infectious diseases, antibiotic usage, industrialization, and a more westernized lifestyle are all considered risk factors (Karimi et al., 2019; Rahmani et al., 2019; Sood et al., 2003). The health status of the Iranian people has improved rapidly over the last three decades, along with the increasing burden of this disease (Shayesteh et al., 2013). Affected patients can experience chronic blood and mucus secretions in the stool in mild cases of the disease to severe bloody diarrhea, bloating, fever, weight reduction, and toxic megacolon (Rao et al., 1988).

Although environmental factors, such as food, are assumed to be responsible for the development and progression of the disease, genetics can only account for 7.5% of the variance of the condition (Geerling et al., 2000). Diet is the most modifiable environmental risk factor for the development of UC; however, the available information in this field is scarce, several studies' theories include its effects on gut microbiota composition, microbial metabolite production, changes in mucosal immunity, and mucosal barrier function (Hekmatdoost et al., 2013; Hou et al., 2011; Khalili et al., 2018). Dairy foods are important dietary sources of fat, protein, and calcium and may be involved in the development of IBD by modulating effects on the gut microbiome and immune response (Da Silva & Rudkowska, 2015). An ecological study showed a strong positive association between milk protein consumption and Crohn's disease (CD) incidence in Japan (Shoda et al., 1996). Clinical and molecular studies suggest that milk nutrients are inversely correlated with mild inflammation and influence cytokines important in the development of IBD, such as tumor necrosis factor‐α (Da Silva & Rudkowska, 2015; Labonte et al., 2013). Furthermore, studies indicated that food allergies in UC patients are primarily caused by an IgE and IgE‐dependent response in the rectal mucosa of the patients (Blanchard et al., 2006). This is because ulcerative colitis patients are now strongly advised to follow a special diet and their main diet is almost a dairy‐free diet (Blanchard et al., 2006). In the 1960s and 1970s, several studies reported that UC patients who eliminated dairy products from their diet improved symptoms and were less likely to have recurring disease activity (Truelove, 1961; Wright & Truelove, 1965). On the other hand, a prospective study of a group of women in France reported no association, which may be due to the small sample size and the risks of CD and UC were not evaluated separately (Jantchou et al., 2010).

These results suggest that dairy may be an important factor in the development or deterioration of ulcerative colitis, although the studies have methodological eliminations (Taxonera & Mendoza, 2004; Wright & Truelove, 1965). Given the inconsistent results of previous studies and the lack of studies in Iran, we decided to investigate the association between total and individual intakes of dietary products and the incidence of UC in Iran.

## 2 METHODS

### 2.1 Design and population

The present case–control study recruited 1122 participants, including 340 UC cases and 782 healthy controls aged >18 years. Participants were selected from three Gastroenterology clinics in Tehran, Iran. The UC presence was confirmed in our cases based on clinical, colonoscopy, and histological examinations within recent six months. The exclusion criteria for both cases and controls included those who suffered from other gastrointestinal diseases than UC, infectious disease, cancer, drug addiction, and pregnancy or breastfeeding. The Shahid Beheshti University of Medical Sciences Ethics Committee gave the study their approval (IR.SBMU.NNFTRI.REC.1401.049). A written informed consent form was given to each participant to sign.

### 2.2 Data collection

For both cases and controls, general questionnaires that were filled out by trained interviewers were utilized to gather information about age, smoking, and alcohol usage. Individuals were divided into two categories in terms of smoking: (1) smokers, including subjects smoking cigarettes or other smoking implements regularly or occasionally and (2) nonsmokers, including past smokers and never smoked. Participants were considered alcoholic if they were habitual alcohol consumers. In order to collect data about physical activity level of participants, the International Physical Activity Questionnaire (IPAQ)‐Short Form was completed for each participant through in‐person interview which has been previously evaluated in Iran in terms of its validity and stability (Moghaddam et al., 2012).

### 2.3 Anthropometric measurements

A trained dietician measured all participants' body weight and height by the use of standardized techniques and calibrated tools (Lee & Nieman, 1996). The body mass index (BMI) was calculated as the ratio of weight (kg) to height squared (square meters) and classified based on World Health Organization guidelines (World Health Organization, 2003).

### 2.4 Dietary intake assessment

A valid and reliable 168‐item semiquantitative food frequency questionnaire (FFQ) was used to assess the dietary intake of participants through face‐to‐face personal interviews (Mirmiran et al., 2010). In both cases and controls, subjects were questioned about their typical food intakes for the year prior to the UC diagnosis and the year prior to the interview. Every participant provided information on how often they consumed each food item on a daily, weekly, or monthly basis. This information was converted to grams per day using home measurements (Ghaffarpour et al., 1999). Since the Iranian food composition table (FCT) is incomplete, both the Iranian and the USDA FCTs were used to determine the daily nutrients and energy intakes (Azar & Sarkisian, 1980; Lan et al., 2023). Milk, cheese, yogurt, and dough (yogurt drink) were included in the study's definition of dairy consumption.

### 2.5 Statistical analysis

Data were analyzed using SPSS software (Version 21.0; Chicago, IL, USA). We used Kolmogorov–Smirnov statistics, *Q*–*Q* plots, and histogram charts to test the normality of all continuous variables. The mean and standard deviation of the mean were computed for representing the continuous variables while the categorical variables were reported as percentages. Using independent Student's *t*‐tests and chi‐squares for continuous and categorical variables, respectively, general features were compared between case and control groups. After dividing participants into three categories according to the tertiles of dairy, milk, yogurt, and cheese consumption, we compared general characteristics and dietary intakes among three categories using one‐way ANOVA for continuous variables and chi‐square for categorical ones. Multiple logistic regression analysis was used to figure out the odds ratios (ORs) and 95% confidence intervals (CIs) for UC based on the number of dairy products eaten per day in each of the tertiles. The reference group was the first tertile. Analyses were adjusted for potential confounders including age, energy, physical activity, alcohol consumption, smoking, intakes of meat, grains, legumes, nuts, fruits, and vegetables, and BMI. There was no collinearity among all the variables in the analysis. The *p*‐value for the trend was determined by employing the tertiles of dairy intake as an ordinal variable within the model. A *p*‐value less than .05 was considered significant and was based on a two‐tailed hypothesis.

## 3 RESULTS

The distribution of general characteristics among UC‐affected and healthy individuals is shown in Table 1. Cases were younger (*p* < .001) and had lower levels of physical activity (*p* < .001). The proportion of individuals with a BMI < 25 (*p* < .001) and alcoholic (*p* = .04) were also significantly higher in the case group. There was no statistically significant disparity observed in terms of other general features between persons affected by UC and those who were healthy.

**TABLE 1:** General characteristics and dietary intakes of cases and controls.

	Cases (*n* = 340)	Controls (*n* = 782)	*p*‐Value^a^
Age (years)	36.5 ± 11.7	43.7 ± 14.5	<.001
Weight (kg)	74.4 ± 15.7	73.5 ± 13.4	.35
Physical activity (MET/min/week)	1048 ± 1264	2059 ± 171	<.001
BMI (kg/m^2^) (%)			
<25	42.4	28.0	<.001
25–29.9	34.1	45.4	
≥30	23.5	26.6	
Males (%)	38.8	40.2	.67
Smoking (smoker) (%)	16.5	18.5	.40
Alcohol (alcoholic) (%)	18	8.7	.04

*Note*: Data are presented as mean (SD) or percent.

Abbreviations: BMI, body mass index; MET, metabolic equivalent.

^a^ Obtained by independent sample *t* test or chi‐square, where appropriate.

Table 2 indicates general characteristics regarding daily consumption of total dairy, milk, yogurt, and cheese tertiles. Participants in the greatest tertile of total dairy intake (>313 g/day) had higher levels of physical activity (*p* < .001), a lower proportion of overweight and obesity (*p* = .02), and a higher male proportion (*p* = .04) compared to those in the lowest tertile. Individuals consuming over 97 g/day of milk were elder (*p* = .01) and more physically active (*p* < .001) than those consuming less than 16 g/day of milk. Compared to the first tertile of yogurt intake (<65 g/day), mean age (*p* = .03), physical activity level (*p* = .003), and overweight and obesity proportion (*p* = .01) were significantly higher in the last tertile of yogurt intake (>138 g/day). Regarding cheese consumption, we observed significant differences in mean age (*p* < .001) and weight (*p* = .04) between the highest (>30 g/day) and lowest (<13 g/day) tertiles.

**TABLE 2:** General characteristics of the study participants cross quintile of dairy, milk, yogurt, and cheese consumption.

	Quintiles of total dairy intake		*p* ^a^	Quintile of milk intake		*p* ^a^	Quintile of yogurt intake		*p* ^a^	Quintile of cheese intake		*p* ^a^
	Quintile1	Quintile5		Quintile1	Quintile5		Quintile1	Quintile5		Quintile1	Quintile5	
Range of intake (g/day)	<118	>395		<8	>164		<33	>227		<8	>30	
Cases/Controls	125/99	38/186		125/90	17/207		117/113	51/182		95/133	79/141	
Age (years)	40.5 ± 12.9	42.6 ± 14.7	.45	42.7 ± 12.9	43.0 ± 15.0	.03	41.3 ± 14.0	43.2 ± 14.3	.10	36.8 ± 12.6	40.4 ± 14.2	<.001
Weight (kg)	73.2 ± 13.8	75.4 ± 14.0	.36	74.8 ± 16.2	73.4 ± 12.6	.75	72.1 ± 13.9	74.6 ± 13.5	.35	72.9 ± 14.3	75.2 ± 14.2	.14
Physical activity (MET/min/week)	1501 ± 1250	1881 ± 549	<.001	1379 ± 854	1625 ± 1328	<.001	1581 ± 1235	1797 ± 605	<.001	1751 ± 1249	1691 ± 821	.74
BMI (kg/m^2^) (%)												
<25	37.1	27.7	.04	34.4	31.7	.12	40.0	27.0	.01	39.9	33.2	.06
25–29.9	34.8	46.9		37.2	45.5		32.2	50.6		36.8	43.2	
≥30	28.1	25.4		28.4	22.8		27.8	22.3		23.2	23.6	
Males (%)	36.6	46.4	.006	37.2	42.9	.77	33.0	39.5	.009	38.2	48.2	.02
Smoking (smoker) (%)	18.3	17	.59	19.1	19.6	.43	17.8	19.3	.88	15.4	22.7	.26
Alcohol (alcoholic) (%)	6.3	9.8	.49	7.4	10.7	.14	7.4	8.2	.78	7.9	8.2	.73

*Note*: Data are presented as mean (SD) or percent.

Abbreviations: BMI, body mass index; MET, metabolic equivalent.

^a^ Obtained by one‐way ANOVA or chi‐square, where appropriate.

The mean (SD) of dietary intakes across tertiles of dairy, milk, yogurt, and cheese consumption is presented in Table 3. Individuals in the uppermost tertile of total dairy consumption, exceeding 313 g/day, exhibited notably elevated levels of energy, proteins, fats, carbohydrates, saturated fatty acid (SFA), calcium, vitamin E, folate, fiber, white meats, red meats, legumes, nuts, fruits, and vegetables. Conversely, their intake of vitamin B6 and refined grains was significantly lower in comparison with those in the lowest tertile of total dairy intake, which fell below 169 g/day. Individuals with higher milk consumption (>97 g/day) had significantly greater dietary intakes of energy, proteins, fats, carbohydrates, SFA, calcium, vitamin E, folate, red meats, legume, fruits, and vegetables, and lower dietary intakes of vitamin B6, fiber, and refined grains than individuals with lower milk consumption (<16 g/day). Participants in the top tertile of yogurt intake (<65 g/day) had significantly higher intakes of energy, proteins, fats, carbohydrates, SFA, calcium, vitamin E, folate, fiber, white meats, red meats, legume, nuts, fruits, and vegetables. Additionally, the intakes of energy, proteins, fats, carbohydrates, calcium, vitamin E, folate, fiber, whole grains, legume, nuts, fruits, and vegetables were significantly higher in the last tertile of cheese intake (>30 g/day). In comparison with subjects in the lowest tertiles, individuals in the highest tertiles of yogurt and cheese consumption had significantly lower intakes of vitamin B6.

**TABLE 3:** Dietary intakes of study participants across quintile of dairy, milk, yogurt, and cheese consumption.

	Quintiles of total dairy intake		*p* ^a^	Quintiles of milk intake		*p* ^a^	Quintiles of yogurt intake		*p* ^a^	Quintiles of cheese intake		*p* ^a^
	Quintile1	Quintile5		Quintile1	Quintile5		Quintile1	Quintile5		Quintile1	Quintile5	
Range of intake (g/day)	<118	>395		<8	>164		<33	>227		<8	>30	
Cases/Controls	125/99	38/186		125/90	17/207		117/113	51/182		95/133	79/141	
Energy (Kcal/day)	2175 ± 735	3130 ± 802	<.001	2435 ± 891	2927 ± 728	<.001	2182 ± 681	2907 ± 834	<.001	2393 ± 916	2859 ± 771	<.001
Nutrients												
Proteins (g/day)	71.3 ± 27.9	121 ± 50.7	<.001	85.6 ± 52.3	110 ± 32.8	<.001	74.3 ± 28.4	112 ± 51.2	<.001	86.4 ± 50.8	103 ± 37.9	<.001
Fats (g/day)	76.8 ± 39.6	119 ± 37.1	<.001	85.1 ± 40.6	111 ± 34.5	<.001	77.5 ± 35.6	108 ± 38.1	<.001	84.7 ± 41.5	108 ± 37.9	<.001
Carbohydrate (g/day)	323 ± 120	450 ± 147	<.001	358 ± 142	430 ± 142	<.001	319 ± 104	423 ± 151	<.001	354 ± 151	411 ± 148	<.001
SFA (g/day)	33.8 ± 52.9	72.6 ± 93.1	<.001	38.7 ± 62.6	69.9 ± 94.7	<.001	33.1 ± 38.0	69.1 ± 94.3	<.001	44.8 ± 77.3	60.4 ± 76.6	.21
Calcium (mg/day)	674 ± 340	1504 ± 406	<.001	872 ± 467	1355 ± 365	<.001	719 ± 337	1396 ± 438	<.001	865 ± 445	1212 ± 432	<.001
Vitamin E (mg/day)	11.2 ± 9.25	19.6 ± 8.41	<.001	11.5 ± 8.65	19.9 ± 7.38	<.001	11.5 ± 8.45	18.3 ± 8.53	<.001	13.5 ± 8.86	16.3 ± 8.81	.001
Vitamin B6 (mg/day)	11.7 ± 10.1	7.72 ± 12.9	<.001	14.7 ± 14.9	4.58 ± 6.66	<.001	11.5 ± 10.8	7.93 ± 12.3	<.001	10.2 ± 12.6	10.1 ± 10.5	.001
Folate (mcg/day)	251.2 ± 303	585 ± 315	<.001	253 ± 324	610 ± 240	<.001	268 ± 292	525 ± 321	<.001	354 ± 335	448 ± 371	<.001
Dietary fiber (g/day)	16.0 ± 8.44	20.5 ± 10.0	<.001	19.1 ± 10.3	18.6 ± 8.27	<.001	15.8 ± 8.13	18.5 ± 9.76	.01	15.5 ± 9.37	18.5 ± 8.36	<.001
Food groups												
Refined grains (g/day)	423 ± 207	395 ± 179	.03	442 ± 242	361 ± 170	<.001	417 ± 214	392 ± 167	.03	424 ± 191	447 ± 205	.05
Whole‐grains (g/day)	100 ± 117	109 ± 94	.15	98.4 ± 108	109 ± 94.1	.12	100 ± 113	99.1 ± 91.6	.93	76.2 ± 70.4	106 ± 111	.002
White meats (g/day)	35.8 ± 33.0	64.8 ± 113.8	<.001	55.4 ± 118	50.4 ± 38.4	.11	40.1 ± 39.9	64.5 ± 111	<.001	53.1 ± 104	54.4 ± 62.7	.03
Red meats (g/day)	25.8 ± 26.3	34.1 ± 24.0	<.001	25.4 ± 26.4	31.3 ± 21.1	.09	28.2 ± 29.4	31.5 ± 22.4	.01	25.5 ± 25.2	32.1 ± 25.1	.03
Legume (g/day)	34.5 ± 30.3	75.3 ± 71.4	<.001	44.6 ± 56.5	65.2 ± 47.6	<.001	36.9 ± 32.1	64.5 ± 66.7	<.001	47.3 ± 52.1	56.7 ± 65.2	.29
Nuts (g/day)	7.66 ± 11.7	13.0 ± 14.2	<.001	9.83 ± 12.6	11.3 ± 12.6	.06	8.08 ± 11.4	11.4 ± 12.0	.005	8.81 ± 10.9	10.8 ± 14.8	.04
Fruits (g/day)	276 ± 188	464 ± 233	<.001	342 ± 226	466 ± 232	<.001	285 ± 180	442 ± 221	<.001	301 ± 225	395 ± 237	<.001
Vegetables (g/day)	273 ± 156	455 ± 230	<.001	330 ± 204	414 ± 201	<.001	277 ± 157	408 ± 209	<.001	312 ± 196	381 ± 198	<.001

*Note*: Data are presented as mean (SD). Comparisons were made using ANOVA.

Abbreviation: SFA, saturated fatty acid.

^a^ Obtained by one‐way ANOVA.

Table 4 shows the estimated crude and adjusted ORs and 95% CIs resulting from multivariable binary logistic regression models concerning the relationship between tertiles of dairy product consumption and UC. In the crude model, a negative association was detected between total dairy intake and the development of UC in the third tertile compared to the first tertile (OR: 0.20; 95% CI: 0.14, 0.28; *p*‐trend < .001). In the multivariable‐adjusted model, after adjustment for potential confounders including age, energy, physical activity, alcohol consumption, smoking, intakes of meat, grains, legumes, nuts, fruits, and vegetables, and BMI, participants in the third tertile of total dairy intake had a greater development of UC compared to those in the lowest tertile (OR: 0.44; 95% CI: 0.24, 0.79; *p*‐trend = .003). Concerning milk consumption, the negative relationship between milk intake and the risk of UC found in the crude model (OR: 0.06; 95% CI: 0.04, 0.10; *p*‐trend .001) remained significant after adjusting for potential confounding variables like age, energy, physical activity, alcohol consumption, smoking, intakes of meat, grains, legumes, nuts, fruits, and vegetables, and BMI (OR: 0.13; 95% CI: 0.07, 0.24; *p*‐trend .001). Moreover, individuals in the top tertile of yogurt consumption had a reduced risk of UC compared to those in the first tertile in the unadjusted model (OR: 0.27; 95% CI: 0.19, 0.38; *p*‐trend < .001). Such relationship was also observed after controlling for potential confounders (OR: 0.52; 95% CI: 0.29, 0.91; *p*‐trend = .02). In the crude or multivariable‐adjusted models, we did not detect any statistically significant relationships between cheese consumption and the risk of UC.

**TABLE 4:** Multivariable‐adjusted odds ratios for ulcerative colitis across quintile of dairy, milk, yogurt, and cheese consumption.

	Quintiles of total dairy intake			*p*‐trend^a^
	Quintile1	Quintile3	Quintile5	
Cases/Controls	125/99	63/162	38/186	
Crude	1	0.30 (0.20, 0.45)	0.16 (0.10, 0.25)	<.001
Model 1	1	0.36 (0.23, 0.55)	0.25 (0.15, 0.41)	<.001
Model 2	1	0.38 (0.22, 0.67)	0.34 (0.18, 0.64)	<.001
Model 3	1	0.40 (0.20, 0.77)	0.40 (0.18, 0.84)	.002
Model 4	1	0.38 (0.20, 0.75)	0.41 (0.19, 0.89)	.003

	Quintiles of milk intake			*p*‐trend^a^
	Quintile1	Quintile3	Quintile5	
Cases/Controls	125/90	51/173	17/207	
Crude	1	0.21 (0.14, 0.32)	0.05 (0.03, 0.10)	<.001
Model 1	1	0.16 (0.10, 0.25)	0.06 (0.03, 0.10)	<.001
Model 2	1	0.22 (0.12, 0.39)	0.11 (0.05, 0.22)	<.001
Model 3	1	0.20 (0.10, 0.38)	0.11 (0.05, 0.25)	<.001
Model 4	1	0.20 (0.10, 0.38)	0.11 (0.04, 0.24)	<.001

	Quintiles of yogurt intake			*p*‐trend^a^
	Quintile1	Quintile3	Quintile5	
Cases/Controls	117/113	55/187	51/182	
Crude	1	0.28 (0.19, 0.42)	0.27 (0.18, 0.40)	<.001
Model 1	1	0.33 (0.21, 0.50)	0.39 (0.25, 0.61)	<.001
Model 2	1	0.34 (0.19, 0.59)	0.40 (0.23, 0.71)	<.001
Model 3	1	0.43 (0.22, 0.82)	0.54 (0.28, 1.04)	.009
Model 4	1	0.45 (0.23, 0.87)	0.58 (0.29, 1.13)	.01

	Quintiles of cheese intake			*p*‐trend^a^
	Quintile1	Quintile3	Quintile5	
Cases/Controls	95/133	56/169	79/141	
Crude	1	0.46 (0.31, 0.69)	0.78 (0.53, 1.14)	.31
Model 1	1	0.63 (0.40, 0.97)	1.32 (0.86, 2.02)	.10
Model 2	1	0.01 (0.48, 0.27)	0.99 (0.57, 1.71)	.78
Model 3	1	0.63 (0.32, 1.22)	1.36 (0.71, 2.61)	.15
Model 4	1	0.62 (0.31, 1.24)	1.34 (0.69, 2.57)	.15

*Note*: Data are presented as OR and 95% CI. Model 1: Adjusted for age and energy. Model 2: Additional adjustment for sex, physical activity, alcohol consumption, and smoking. Model 3: Additional adjustment for meat, grains, legumes, nuts, fruits, and vegetable intake. Model 4: Additional adjustment for BMI.

^a^ Obtained from binary logistic regression.

## 4 DISCUSSION

In this large‐scale case–control study, greater total dairy intake decreased odds of UC. As for specific dairy products, our observations demonstrate an inverse link between milk and yogurt intakes and UC and a null association between cheese consumption and this condition. This is among the first studies investigating such associations.

Initially considered a disease in Western countries (Windsor & Kaplan, 2019), the prevalence of UC is growing day by day in Middle Eastern countries (Mosli et al., 2021) including Iran (Olfatifar et al., 2021; Safarpour et al., 2013), imposing a significant financial strain on society and the healthcare system (Beard et al., 2020). Mountains of evidence showed a significant role of diet as an important risk factor for this inflammatory disorder (Ananthakrishnan et al., 2015; Andersen et al., 2012; Chapman‐Kiddell et al., 2010). Evaluated consumption of red and processed meats, sugar, and trans‐unsaturated fatty acids seemed to contribute to the increased prevalence of this condition (Ananthakrishnan et al., 2014; Dong et al., 2022; Katschinski et al., 1988; Sakamoto et al., 2005). Much less, vegetables and fruits probably due to their high fiber content can lower odds of UC (Hansen et al., 2011; Milajerdi et al., 2020). However, in this study, we tried to examine dairy intake in relation to UC. We discovered a link between total dairy consumption and a lower risk of UC. Likewise, in another case–control study in Iran, researchers reported both high‐ and low‐fat dairy products to be marginally protective against UC (Farsi et al., 2022). In contrast to our observations, dairy product intake did not change odds of UC in another study in a small study examining dietary protein intakes and risk of UC (Rashvand et al., 2015). In addition, multicenter case–control research by Sakamoto et al. (2005) found no association between milk and dairy products and the likelihood of developing UC. Additionally, total dairy products were not linked with UC in a prospective cohort research done by Opstelten et al. (2019). In the present study, we observed that milk consumption might reduce UC risk. Evidence across studies is conflicting; results of the EPIC cohort demonstrated no link between milk and UC, although interestingly milk consumption reduced risk of UC development in patients who were diagnosed more than 3 years after enrolment (Opstelten et al., 2019). In addition, in a case–control study, milk intake was not associated with UC risk (Maconi et al., 2010), while yogurt intake lowered UC chances in this study. Similar to our finding, in a retrospective study in two European cohorts, daily yogurt intake reduced IBD incidence (Preda et al., 2020). However, another cohort study reported no significant association between yogurt consumption and UC development (Opstelten et al., 2019).

Lastly, we found no association between cheese intake and UC risk, the same findings were observed in a population‐based case–control study (Maconi et al., 2010), and a cohort study (Opstelten et al., 2019). The diversity of dairy products, insufficient study power, different methods for assessment of dietary intake, and other methodological limitations may to some extent explain the inconsistencies in the findings of these studies. Prospective cohort studies are needed to come to a conclusion.

Although we are not sure how exactly dairy product intake might be protective against UC, there are some possible mechanisms. The beneficial effect of milk and other dairy products might be because of their rich content of calcium. This micronutrient can bind with proinflammatory secondary bile acids and reduce intraluminal inflammation (Aune et al., 2012; Norat & Riboli, 2003). Calcium also has been found to inhibit the tumor necrosis factor‐α (TNF‐α) pathway and therefore suppress IBD in mice (Zhu et al., 2005). Considering the role of intestinal epithelial cells in the pathogenesis of IBD and UC (Okamoto & Watanabe, 2016), calcium intracellular action which is to prevent proliferation of gut epithelial cells through inducing their differentiation (Norat & Riboli, 2003) might contribute to gut health. Moreover, dairy products have been found to reduce inflammation (Labonté et al., 2013; Nieman et al., 2021; Ulven et al., 2019) through anti‐inflammatory properties of vitamin D (Guillot et al., 2010; Mouli & Ananthakrishnan, 2014) in fortified dairy products or even by some milk‐driven proteins like lactoferrin (Bharadwaj et al., 2010). On the other hand, dairy products especially fermented ones such as yogurt, due to their probiotic properties, can shape gut microbiome which, in turn, regulates innate immune response and inflammation (Aune et al., 2012; Bordoni et al., 2017; Santos Rocha et al., 2012; Shin et al., 2002; Veiga et al., 2010). Interestingly, in the present study, although we found all dairy products to reduce UC risk, failed to observe any significant link between cheese intake and this disorder. Cheese is considered a full‐fat dairy product (Hu et al., 2022), and therefore rich in saturated fatty acids (SFAs) so its beneficial effects such as its probiotic properties and those correlated to high calcium content of this product can be modulated because of proinflammatory properties of SFAs (Devkota et al., 2012).

This case–control study presents several strengths. Applying a validated and reliable FFQ, adjustments for several potential covariates, and large sample size are among these strengths. Although some limitations must be considered when interpreting the results, first, like all observational studies, we cannot conclude causality. Secondly, even a well‐designed case–control study is still subjected to recall and selection biases. Finally, the possible effects of several unmeasured or residual confounding factors cannot be disregarded.

In conclusion, we observed that higher consumption of total dairy products might decrease the odds of UC. To be specific, milk and yogurt are inversely associated with this disorder. However, no link was found between cheese intake and UC. Longitudinal observational studies especially cohorts are needed to further assess these associations.

## AUTHOR CONTRIBUTIONS

**Mohammad Reza Amini:** Formal analysis (equal); writing – original draft (equal); writing – review and editing (equal). **Zeinab Khademi:** Writing – original draft (equal). **Marieh Salavatizadeh:** Writing – original draft (equal). **Zahra Kalantar:** Writing – original draft (equal). **Nasser Ebrahimi‐Daryani:** Data curation (equal). **Ahmad Esmaillzadeh:** Conceptualization (equal); supervision (equal). **Azita Hekmatdoost:** Conceptualization (equal); supervision (equal); writing – review and editing (equal).

## FUNDING INFORMATION

This work was supported financially by the National Nutrition and Food Technology Research Institute, Shahid Beheshti University of Medical Sciences, Tehran, Iran (43003016).

## CONFLICT OF INTEREST STATEMENT

The study's authors affirm that there were no financial or commercial ties that might be viewed as having a potential conflict of interest.

## ETHICS STATEMENT

Shahid Beheshti University of Medical Sciences assessed and granted approval for investigations involving human subjects (IR.SBMU.NNFTRI.REC.1401.049). In order to take part in this study, the patients/participants gave their written informed consent.

## Data Availability

The data that support the findings of this study are available on request from the corresponding author. The data are not publicly available due to privacy or ethical restrictions.
